# Psychopathology and cognitive deficits in young people exposed to complex trauma

**DOI:** 10.1192/bjo.2021.149

**Published:** 2021-06-18

**Authors:** Stephanie J Lewis, Karestan C Koenen, Antony Ambler, Louise Arseneault, Avshalom Caspi, Helen L Fisher, Terrie E Moffitt, Andrea Danese

**Affiliations:** 1 Institute of Psychiatry, Psychology & Neuroscience, King's College London, South London and Maudsley NHS Foundation Trust; 2 Harvard TH Chan School of Public Health; 3 Institute of Psychiatry, Psychology and Neuroscience, King's College London; 4 Duke University, Institute of Psychiatry, Psychology and Neuroscience, King's College London

## Abstract

**Aims:**

Complex traumas are traumatic experiences that involve multiple interpersonal threats during childhood or adolescence, such as repeated abuse. This type of trauma is hypothesized to lead to more severe psychopathology and poorer cognitive function than other non-complex traumas, such as road traffic accidents. However, empirical testing of this hypothesis has been limited to clinical or convenience samples and cross-sectional designs. To better understand this topic, we aimed to investigate psychopathology and cognitive function in young people exposed to complex, non-complex, or no trauma from a population-representative longitudinal cohort, and to consider the role of pre-existing vulnerabilities.

**Method:**

Participants were from the Environmental Risk (E-Risk) Longitudinal Twin Study, a population-representative birth-cohort of 2,232 children born in England and Wales in 1994-95. At age 18 years (93% participation), we assessed lifetime exposure to complex and non-complex trauma. We also assessed past-year psychopathology including general psychopathology ‘p’ and several psychiatric disorders, as well as current cognitive function including IQ, executive function, and processing speed. Additionally, we prospectively assessed early childhood vulnerabilities including internalizing and externalizing symptoms at age 5, IQ at age 5, family history of mental illness, family socioeconomic status, and sex.

**Result:**

We found that participants who had been exposed to complex trauma had more severe psychopathology and poorer cognitive function across wide-ranging measures at age 18, compared to both trauma-unexposed participants and those exposed to non-complex trauma. Early childhood vulnerabilities had an important role in these presentations, as they predicted risk of later complex trauma exposure, and largely explained associations of complex trauma with cognitive deficits, but not with psychopathology.

**Conclusion:**

By conflating complex and non-complex traumas, current research and clinical practice under-estimate the severity of psychopathology and cognitive deficits linked with complex trauma, as well as the role of pre-existing vulnerabilities. A better understanding of the mental health needs of people exposed to complex trauma and underlying mechanisms could inform the development of new effective interventions.

